# Diagnostic and Therapeutic Maneuvers for Anterior Canal BPPV Canalithiasis: Three-Dimensional Simulations

**DOI:** 10.3389/fneur.2021.740599

**Published:** 2021-09-24

**Authors:** Anita Bhandari, Rajneesh Bhandari, Herman Kingma, Michael Strupp

**Affiliations:** ^1^Vertigo and Ear Clinic, Jaipur, India; ^2^NeuroEquilibrium Diagnostic Systems Pvt Ltd., Jaipur, India; ^3^Department of Otorhinolaryngology, Head and Neck Surgery, Maastricht University Medical Centre, Maastricht, Netherlands; ^4^Faculty of Physics, Tomsk State University, Tomsk, Russia; ^5^Department of ENT, Aalborg University, Aalborg, Denmark; ^6^Department of Neurology and German Center for Vertigo and Balance Disorders, Ludwig Maximilian University of Munich, Munich, Germany

**Keywords:** BPPV, anterior canal, simulation, maneuvers, Yacovino, reverse Epley, short canal repositioning maneuver

## Abstract

**Background and Objectives:** Anterior canal BPPV is a rare BPPV variant. Various diagnostic and therapeutic maneuvers have been described for its management. The aim of this study was to use three-dimensional simulation models to visualize otoconial debris movement within the anterior canal during diagnostic tests and different liberatory maneuvers. This can help to optimize existing treatment maneuvers and help in the development of better management protocols.

**Methods:** Based on reconstructed MRI images and fluid dynamics, a 3D dynamic simulation model (as a function of time) was developed and applied. Simulations of the supine head-hanging test for diagnosis of ac-BPPV were studied. Three repositioning maneuvers were simulated: 1) the Yacovino maneuver and its modifications, 2) the reverse Epley maneuver and 3) the short canal repositioning (CRP) maneuver.

**Results:** The simulation showed that the supine head-hanging test is a good test for diagnosis of ac-BPPV affecting both labyrinths and demonstrated why there is no inversion of nystagmus on sitting up. The Yacovino maneuver was seen to be an effective treatment option for ac-BPPV without having to determine the side involved. However, simulations showed that the classical Yacovino maneuver carried a risk of canal switch to the posterior canal. To overcome this risk, a modified Yacovino maneuver is suggested. The reverse Epley maneuver was not an effective treatment. Short CRP is useful in ac-BPPV treatment; however, it requires determination of side of involvement.

**Conclusion:** The 3D simulator of the movement of the otoconial debris presented here can be used to test the mechanism of action and the theoretical efficacy of existing diagnostic tests and maneuvers as well as to develop new treatment maneuvers to optimize BPPV treatment.

## Introduction

**Figure 1:**
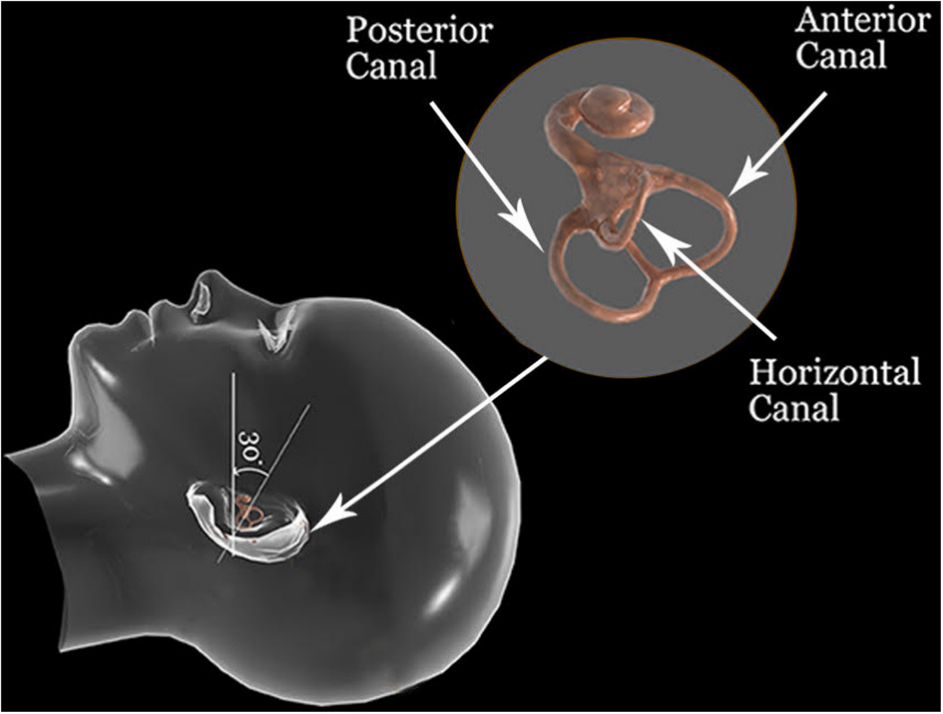
Anatomical orientation of the semicircular canals in a supine head position.

Anterior canal BPPV (ac-BPPV) was first described in 1987 (1). It is considered the rarest form of semicircular canalolithiasis (2). Two factors may explain its low incidence: The anterior canal is situated in the superior position of the labyrinth with the non-ampullary arm of the canal descending directly into the common crus and onward into the vestibule (Figure 1). The anterior canal is higher than both the posterior and horizontal canals. This anatomical position makes it less likely for the otoconial debris to enter the canal against gravity (3). Furthermore, this anatomical orientation should also facilitate self-clearance of the otoconial debris due to gravity (4). The low incidence might be one of the major reasons for the paucity of studies and literature describing this clinical entity, which the Barany Society Consensus document still calls an emerging and controversial entity (5).

### Diagnostic Maneuvers

In addition to its low incidence, there are many ambiguous issues in terms of the diagnosis and the treatment (see below) of ac-BPPV. The positional tests described for diagnosis are the Dix–Hallpike and supine head-hanging tests. ac-BPPV is characterized by a vertical downbeat nystagmus with a torsional component toward the affected side (5) evoked by the Dix–Hallpike and supine head-hanging tests. However, the torsional component is not always clear and is less intense than the vertical one and, hence, needs to be differentiated from posterior canal down-beating BPPV (6, 7). Therefore, determining the affected side based on the Dix–Hallpike examination can often be difficult, thus, further complicating proper diagnosis and treatment (8–10). The supine head-hanging test is considered to be a more sensitive test for ac-BPPV as it acts in the sagittal plane and, thus, stimulates both anterior canals at the same time (5, 10, 11). However, there is, so far, no generally accepted diagnostic maneuver for ac-BPPV. Down-beat nystagmus on positional tests can be associated with central disorders and should be excluded from peripheral down-beating nystagmus (12).

### Therapeutic Maneuvers

Various therapeutic maneuvers have been described for the treatment of ac-BPPV. Considering the posterior and anterior canals as co-planar, reversal of maneuvers used for posterior canal-BPPV treatment, such as the Epley and Sémont maneuvers, were recommended to treat ac-BPPV (13–15). The reversed maneuver is started from the healthy side. The Yacovino maneuver was proposed as a treatment option with the distinct advantage that the side of involvement does not need to be identified for treatment (16). The short canal repositioning maneuver (short CRP maneuver) works on the basis of a modified form of the Epley maneuver, which can be used in the treatment of ac-BPPV after determining the side of involvement (6). Various other maneuvers described in literature, which require identification of the side of involvement, have been described (17–20). Based on the orientation of the canal during these maneuvers and the underlying biomechanics, each maneuver theoretically has its advantages and disadvantages similar to treatment maneuvers for posterior and horizontal canal BPPV (4, 16, 21, 22).

### Simulation of the Maneuvers

Many two-dimensional illustrations for BPPV have been described, but they have the limitation of providing the view from only one angle and showing only the initial and final position of the debris. In this article, we present the simulation of ac-BPPV in the three-dimensional space to optimally visualize the movement of the head, labyrinth, and otoconial debris for practical clinical use. We used a software-based simulator (4, 21, 23) to study different positional tests and liberatory maneuvers in ac-BPPV by demonstrating the continuous dynamic movement of the otoconial debris in the anterior canal as a function of time and angulation. The simulation depicts the movement of the debris in the canal at each step. It is important to note that these are true simulations of the debris movement based on the biophysics of BPPV and not simple animations. Basic assumptions regarding the debris size and distribution, endolymph viscosity, and canal geometry have been taken into consideration (4, 23).

In this study, we specifically used simulations of (a) the supine head-hanging test for the diagnosis of ac-BPPV, (b) the Yacovino maneuver (16) and its modifications for the treatment of ac-BPPV, (c) the Epley maneuver done from the opposite side (reverse maneuver), and (d) the short CRP maneuver (6). The aim of the simulations was to find out which maneuver might theoretically work, which does not, and which one might even be superior. These findings can also be the basis of a controlled trial for the diagnosis and treatment of ac-BPPV.

## Methods

Based on reconstructed MRI images and fluid dynamics, a 3D dynamic simulation model (as a function of time) was developed and applied [for more details, refer to Bhandari et al. (23)] The simulation allowed placement of the debris at variable positions within the canal and also in more than one canal simultaneously. The time between the two steps of the maneuver required for the debris to reach the desired position is accelerated for better understanding and ease of demonstration.

## Results

The results of the simulations of the following maneuvers will be presented: for the diagnosis of ac-BPPV, the supine head-hanging test; for its treatment, the Yacovino maneuver and its modifications, the Epley maneuver done from the opposite side (reverse maneuver), and the “short CRP maneuver.”

### Diagnostic Maneuvers

#### Supine Head-Hanging Test for the Diagnosis of Anterior Canal BPPV

Clinically, ac-BPPV is characterized by a vertical downbeat nystagmus with a torsional component toward the affected side when the individual is looking straight ahead as evoked by the supine head-hanging test. There is usually no inversion (see below) of the downbeat nystagmus on returning to the sitting position.

The simulation shows that in the deep head-hanging position, there is ampullofugal movement of the debris, which leads to an excitation of the anterior canal (Simulation 1 in Supplementary Material). This causes a downbeat nystagmus with torsion toward the side of involvement when the individual looks straight ahead. This implies that the supine head-hanging test is useful for the diagnosis of both anterior canals. In the second step, when the subject comes back to the sitting position, the debris moves further toward the utricle (continuing the ampullofugal movement) and not back toward the ampulla. This explains why there is no inversion of nystagmus when the subject returns to sitting position and the natural remission. Simulation 1 in Supplementary Material.

### Treatment Maneuvers

#### Yacovino Maneuver, New Modified Yacovino Maneuver, and Failed Anterior Canal Maneuver

We studied two types of these maneuvers using the simulator: the original Yacovino maneuver (16) and a new modified Yacovino maneuver, which—as will be shown below—has a lower risk of a transition from anterior canal to posterior canal BPPV, based on our simulation.

The original Yacovino maneuver consists of four steps each performed at an interval of 30 s as the otoconia moves down about 1% of the diameter of the canal per second under the influence of the gravity acting on it (24, 25). The four steps are as follows: step 1: sit straight; step 2: bring to the head to the head-hanging position, 30° below the horizontal plane; step 3: head is elevated so that the chin touches the chest; and step 4: back to the sitting position.

As the anterior canal lies in the vertical plane, the head should remain straight on starting the maneuver. In the next step, the head of the subject is taken down to 30° below the horizontal plane. This inverts the anterior canal such that the ampullary arm lies at the most superior position, whereas the non-ampullary arm is placed inferiorly. The otolith debris move ampullofugally to reach the most dependent position in the canal. Next, the subject is taken to the chin-to-chest position. This takes the debris further ahead in the canal. However, the simulation shows that at this point, there is a risk that the debris enters the posterior canal, leading to a canal switch. In the final step, the subject sits up and bends the head forward, leading to the debris being repositioned to the utricle. Simulation 2 in Supplementary Material.

If the subject is kept for a longer duration in the chin-to-chest position, there is an even higher risk of the debris entering into the posterior canal. This can be seen in Simulation 3 in Supplementary Material.

To avoid the risk of canal switch, we propose a modification of the Yacovino maneuver. In this variation, the subject is brought directly from the head-hanging position to the sitting position. After an interval of 30 s, the neck of the subject is flexed forward at an angle of 45°. Simulation 4 in Supplementary Material shows that this modification brings a better repositioning of the otoconial debris into the utricle. The chin-to-chest position has been omitted to avoid the risk of moving the debris from crus commune to posterior canal. Simulation 4 in Supplementary Material.

Correct angulation of the head and waiting in between each step of the maneuver is important to allow the debris to move further in the canal. Simulation 5 in Supplementary Material demonstrates how incorrect head angulation and inadequate time between steps can lead to failure of treatment by the maneuver. In this case, the subject is moving from the head-hanging position to the sitting position and then immediately bending the neck on sitting. The simulation demonstrates that the debris falls back toward the ampulla instead of moving toward the utricle, thus, leading to a failed repositioning. Simulation 5 in Supplementary Material.

### Reverse Epley Maneuver

As the ipsilateral anterior and contralateral posterior canals are co-planar, repositioning maneuvers used for pc-BPPV treatment have been advocated for ac-BPPV treatment as well. In this way, an Epley maneuver is performed from the right side for repositioning of left ac-BPPV and vice versa, i.e., a reverse maneuver (13, 14). Simulation 6 in Supplementary Material demonstrates that the reverse Epley maneuver is theoretically not very effective as there is a high risk that the debris moves backward and falls back toward the ampulla instead of moving toward the utricle. Simulation 6 in Supplementary Material.

### “Short Canal Repositioning Maneuver”

The short CRP maneuver (6) or short Epley was proposed to improve the results of the classic repositioning maneuvers in ac-BPPV treatment. The steps for this maneuver are step 1: seated upright with head turned to the affected side by 45°; step 2: head-hanging position with the head 40° below the horizontal; step 3: while still in the head-hanging position, the head is turned to the healthy side; and step 4: back to sitting position.

This maneuver is similar to the classic Epley maneuver with the variation of omitting the step of turning to the nose-down position to the healthy side. This modification facilitates progressive movement of the debris out of the canal. Simulation 7 in Supplementary Material shows that short canal repositioning is an effective treatment option for ac-BPPV. However, it requires determination of the side of involvement, as in the reverse Epley maneuver. Simulation 7 in Supplementary Material.

In this simulation, it was seen that the 30° head hanging position is as effective as the 40° angulation described by the authors. This shows that increasing the angle of the head beyond 30° does not influence treatment outcome.

## Discussion

BPPV involving the anterior canal has a low incidence. However, its low incidence contrasts with the clinical importance of its most prominent characteristic, positional downwardly beating nystagmus, which also occurs as central positional nystagmus associated with various brainstem and cerebellar lesions, and may indicate a sinister pathology (26). In contrast to BPPV affecting the other canals, data on the diagnostic techniques and therapeutic maneuvers for ac-BPPV are sparse.

The major findings of this study using software simulations are as follows:

1. Diagnostic tests for ac-BPPV—the supine head-hanging test is an effective diagnostic test for ac-BPPV in which both canals can be tested together.
2. Therapeutic maneuvers: (a) The treatment outcome of the Yacovino maneuver can be improved with a modification in steps as demonstrated in the new “simplified Yacovino maneuver”; (b) the reverse Epley maneuver is not an effective treatment option; and (c) the short CRP maneuver is a useful treatment option; however, it requires the determination of the side of involvement.

### Diagnostic Tests for Anterior Canal BPPV

The Dix–Hallpike maneuver and the supine head-hanging test have been described as the positional tests to diagnose ac-BPPV. There are, however, conflicting reports regarding which side the Dix–Hallpike test generates stronger nystagmus—ipsilateral, contralateral, or both (1, 6, 8, 26). These reports indicate that the results from the D-H examination may vary in different patients. The Bárány Society has classified ac-BPPV canalithiasis (5) as positional nystagmus elicited by the Dix–Hallpike maneuver (on one or both sides) or in the supine straight head-hanging position. The nystagmus beats predominantly vertically downward in the Dix Hallpike position, and nystagmus may be stronger or exclusively present with the affected ear up or down.

Based on our simulations, the supine head-hanging test seems to be a more suitable positional test for the anterior canals as it aligns the parasagitally placed canals closest to the mid-sagittal plane (22). Keeping the head in a non-rotated position is more beneficial for movement of debris within the anterior canal compared with the rotated position of the Dix–Hallpike maneuver. Furthermore, as the head reaches a lower position in the supine head-hanging test compared with the Dix Hallpike maneuver, the effect of gravity on the debris in the canal will be enhanced. The angle of the ASC relative to the earth-horizontal is approximately 20° larger during the straight head hanging position than during the D-H test (8, 27). The simulation model demonstrated that the otoconial debris in ac-BPPV affecting either side would move ampullofugally in the canal during the supine head-hanging test.

### Reversal of the Nystagmus

Most positioning tests show a reversal of nystagmus on returning to the initial position. ac-BPPV is characterized by vertical downwardly beating paroxysmal nystagmus evoked by the supine head-hanging test without inversion of the down-beating vertical nystagmus on returning to the sitting position. This can be explained by the fact that the SHH test inverts the ac to allow debris to reach the peak of the ac, and then, upon returning the patient to the sitting position, allows it to migrate further into the common crus (1). Toward the end of the SHH, if the otoconia debris traverses the common crus, the pressure field of the moving otoconia is exerted across both the anterior and posterior canals and the direction of the nystagmus is affected accordingly (8). Simulation 1 in Supplementary Material shows the debris moving from the ampullary arm at the beginning of the test to the lowest position of the canal in the head-hanging position. The lowest position is actually the most superior part of the ac. When the subject is brought back to the sitting position, the debris moves further ampullofugally in the same direction. Hence, the nystagmus trajectory will remain the same. This finding is in agreement on the statement that both ac and apogeotropic posterior canal BPPV are characterized by paroxysmal nystagmus evoked in different positions and rarely inverting when returning to the sitting position (1).

However, this is in contrast to what was reported in some studies where the authors report that on returning to the sitting position, there should be a less intense nystagmus in the opposite direction, that is, upbeating with the torsional component beating away from the affected ear (1, 2, 11). Thus, we see that when returning to the sitting position some authors have described a lack inversion of the down-beating vertical nystagmus (1, 17), while others described it with an inversion (2, 11, 17, 22). Therefore, this has to be re-evaluated in clinical studies.

### Treatment Maneuvers for Anterior Canal BPPV

As a general rule in BPPV, there is only one optimal geometry to maneuver debris in a particular canal (11), and all maneuvers attempt to bring the debris around a circle of the affected canal. For treatment of ac-BPPV, the anterior canal is positioned upside-down to allow debris to fall to the “top” of the canal, and then further steps prompt the debris to further migrate into the common crus and then into the vestibule. Various attempts to modify maneuvers often lead to another unique way to accomplish the same goal of particle repositioning (6). Several maneuvers have been described and recommended for ac-BPPV, but there is, so far, no consensus on its best treatment. Our simulation has evaluated the pros and cons of these maneuvers, which will have clinical implications.

#### Yacovino Maneuver and the New Modified Yacovino Maneuver Based on our Simulation

Both utilize the principle of gravity to move the debris through the canal back into the utricle (16). This maneuver has the distinct advantage over other maneuvers in that the determination of the side of involvement is not a pre-requisite. The Yacovino maneuver involves taking the patient to the supine head-hanging position, followed by flexing the neck to the chin-to-chest position and then bringing the patient up to the sitting position, finally bending the neck. Simulation 2 in Supplementary Material shows how the original Yacovino maneuver is effective in treating ac-BPPV. In this simulation, it was also demonstrated that in the chin-to-chest position, there is a chance of the debris entering into the posterior canal, resulting in canal switch instead of repositioning to the utricle. In fact, if the patient is kept in this chin-to-chest position for a longer time, Simulation 3 in Supplementary Material shows that the chances of canal switch increases. Canal switch is a complication of CRP where the debris moves from one canal to another. It is most commonly described for posterior canal BPPV converting to the superior or horizontal canal (28, 29). The classification of ac-BPPV (1) includes canal conversion to the posterior canal during or immediately after the therapeutic manoeuver as “certain” evidence of ac-BPPV. Studies have shown canalar conversion from anterior canal into typical posterior canal BPPV after Yacovino maneuver which required additional maneuvers (*two-step therapy*) (1). The Yacovino maneuver can result in uncontrolled conversions into a PC-BPPV after performing the maneuver (1, 22). All semicircular canals could be affected by free-moving otoconia, and an iatrogenic canal switching during CRM is possible (30).

To solve this problem in the classic Yacovino maneuver, we propose a modification to make the maneuver simpler and theoretically more efficient. Simulation 1 in Supplementary Material showed that in the supine head–hanging position, the debris reach the apex of the canal and in the sitting position, the debris move further ahead in the canal rather than falling back to the ampulla. As we mentioned before, this is why inversion of nystagmus does not occur in the supine head-hanging test. Taking this fact into consideration, we have proposed a modification of the Yacovino maneuver. In this modified maneuver shown in Simulation 4 in Supplementary Material, after the supine head-hanging position, the subject is taken immediately from supine head deep hanging 30° below the horizontal to the sitting position. After waiting for 30 s in the sitting position, the neck of the subject is flexed. The simulation shows that the debris reaches the highest point of the ac under the influence of gravity in the supine head-hanging position. When the subject goes to the sitting position next, the debris travels further ahead through the crus commune and onward to the utricle. The final step of bending the neck prevents the repositioned debris from re-entering into the ac.

### Timing

One of the most critical factors to achieve successful repositioning is to allow adequate time between the two steps of the maneuver so that the particle reaches the lower most position of the canal, due to gravity before moving to the next step (5, 6). An insufficient waiting period between the steps does not allow gravity to take the particle to the required position. In Simulation 5 in Supplementary Material, when the neck is bent immediately without waiting for the particle to come to the lowermost position, the otolith debris fails to move toward the common crus and instead falls backward toward the ampulla. This underlines the importance of waiting between each step of the maneuver for the debris to reach the most dependent position. Although the minimum time interval between the two steps is not fixed, we propose 30 s between each step or till the induced nystagmus subsides. Yacovino maneuver was subsequently re-described with subtle differences: a 3-min pause in each position rather than 30 s, and rapid transitions (31). However, we recommend a 30-s interval between steps as longer waiting time may encourage canal switch and rapid transition may result in inadequate debris progression.

#### “Reverse Epley Maneuver”

This was one of the first repositioning maneuvers proposed for the treatment of ac-BPPV (14, 32, 33). Various studies have shown the efficacy of this maneuver to treat ac-BPPV (1, 16, 24, 26); however, detailed data on the number and the history of the patients, as well as the outcome of this treatment are lacking (8). In the reverse Epley maneuver, the head is dropped into the Dix-Hallpike position with the affected ear up and the patient is then moved in 90° steps toward the unaffected side as in the CRP (10). Thus, the same positioning sequence as for the contralateral posterior canalithiasis is performed. The geometry of the ac is such that one would expect this maneuver could even make it worse because it involves nose-down positioning (11). Simulation 6 in Supplementary Material demonstrates the Epley maneuver performed for a contralateral ac-BPPV. It is seen that turning the head by 45° to the healthy side and going down by 30° brings the debris ampullofugally to the lowest position. Turning the head to the affected side by 90° takes the debris to the apex of the canal. When the subject is further turned by 90° to the nose pointing down position, this leads to retrograde movement of the debris toward the ampulla. This brings us to the conclusion that the “reverse Epley” is evidently not effective for the treatment of ac-BPPV.

#### “Short Canal Repositioning Maneuver”

To overcome the drawback of the “reverse Epley,” a modified maneuver called the “short canal repositioning maneuver” was proposed (6). It also requires determination of the side of involvement. After determination of the side, the head of the subject is turned by 45° to the affected side and taken to the head hanging position. An enhancement of hanging the head to lower than 30° in this position was described to promote more definite progression of the otolith mass around the circumference of the canal. In the next step, the subject's head is turned to the healthy side by 90°. Then the subject is brought back to the sitting position (the nose pointing down position of the classic Epley maneuver has been omitted). Simulation 7 in Supplementary Material shows the “short CRP” to be an effective treatment option for ac-BPPV. The simulation also showed that 30° head hanging is sufficient to help the debris progress through the canal and an increase in the angle may not really be required.

The past few decades have increased our knowledge about BPPV; however, some aspects are still not understood or are controversial (34–36). Perhaps, answers will come when we can image the material in the semicircular canals and see its motion (2). Until direct imaging of debris becomes possible, the 3D simulations provide a useful tool to understand the changing orientation of the semicircular canals with changes in head positions and angulations. This tool can aid in optimizing treatment modules.

## Limitations

Our study is based on the orientation of the semicircular canals obtained from the reconstructed MRI images. However, the orientation of the canals varies from one patient to another. Different morphology and orientation of the canals are an important factor for the success or failure of a repositioning maneuver. The major limitation of our study is that it fails to represent the complete population due to these variables. The simulations we have used do not take into account the impact of different debris sizes and the possibility that the debris can be located in different parts of the canal at the same time; issues that may differ from patient to patient. This is not implemented as there are many unknown variables and visualizing the otolith movement for each and every patient is beyond the scope of our study. Despite these limitations, the simulators provide an effective detailed understanding about the mechanism of the maneuvers and conclude which therapeutic maneuver could be most effective.

## Conclusion

These simulations show that the new simplified Yacovino maneuver is an effective treatment option for ac-BPPV. It also reduces the risk of canal switch, which may occur in the original Yacovino maneuver. In both, there is no need to determine the affected side as required in the short CRP and the (theoretically not effective) reverse Epley maneuvers.

On the basis of our findings, we encourage a clinical validation of our theoretical results, i.e., randomized controlled clinical trials directly comparing the efficacy of the various maneuvers discussed here.

## Data Availability Statement

The original contributions presented in the study are included in the article/Supplementary Material, further inquiries can be directed to the corresponding author/s.

## Author Contributions

AB conception and development of 3D simulation, formulated study design and interpreted data, and written the manuscript. RB conception and development of the software for 3D simulation, contribution of the study design, and interpretation of the data. HK development of simulation models, improvement in clot movement and visualization, and optimization of text. MS conception of the study, contribution of the study design, interpretation of the data, and drafting and editing of the manuscript for intellectual content. All authors reviewed and approved the manuscript.

## Conflict of Interest

RB is employed by NeuroEquilibrium Diagnostic Systems Pvt Ltd, India. The remaining authors declare that the research was conducted in the absence of any commercial or financial relationships that could be construed as a potential conflict of interest.

## Publisher's Note

All claims expressed in this article are solely those of the authors and do not necessarily represent those of their affiliated organizations, or those of the publisher, the editors and the reviewers. Any product that may be evaluated in this article, or claim that may be made by its manufacturer, is not guaranteed or endorsed by the publisher.

## Supplementary Material

Simulation 1 Supine head-hanging test.

Simulation 2 Yacovino maneuver.

Simulation 3 Canal switch in Yacovino maneuver.

Simulation 4 Modified Yacovino maneuver.

Simulation 5 Failed anterior canal maneuver.

Simulation 6 Reverse Epley maneuver.

Simulation 7 Short CRP maneuver.
